# Workplace Interventions for Type 2 Diabetes Mellitus Prevention—an Umbrella Review

**DOI:** 10.1007/s11892-023-01521-3

**Published:** 2023-09-20

**Authors:** Katarzyna Wnuk, Jakub Świtalski, Tomasz Tatara, Wojciech Miazga, Sylwia Jopek, Anna Augustynowicz, Urszula Religioni, Mariusz Gujski

**Affiliations:** 1 School of Public Health, Centre of Postgraduate Medical Education of Warsaw, 01-826 Warsaw, Poland; 2 Department of Health Policy Programs, Department of Health Technology Assessment, Agency for Health Technology Assessment and Tariff System, 00-032 Warsaw, Poland; 3 Department of Health Economics and Medical Law, Faculty of Health Sciences, Medical University of Warsaw, 01-445 Warsaw, Poland; 4 Department of Public Health, Faculty of Health Sciences, Medical University of Warsaw, 02-091 Warsaw, Poland

**Keywords:** Diabetes mellitus, T2DM, Workplace, Health promotion

## Abstract

**Purpose of review:**

Type 2 diabetes mellitus (T2DM) is a chronic disease that may lead to severe complications. The main methods of preventing or delaying the onset of T2DM include lifestyle changes. The purpose of this study is to identify and evaluate the effectiveness of workplace interventions aimed at preventing type 2 diabetes. An umbrella review was conducted in accordance with the Cochrane Collaboration guidelines. Searches were performed in Medline (via PubMed), Embase (via OVID), and Cochrane Library databases. The quality assessment of the included studies was performed using the AMSTAR2 tool.

**Recent findings:**

The final analysis included 7 studies. The majority (4 of 7) of the studies included in the review focused on workplace interventions based on the guidelines of the US Diabetes Prevention Program (DPP) or other similar programs. The method of decreasing the risk of type 2 diabetes among employees are programs consisting of multiple approaches aimed at improving parameters associated with diabetes, i.e., body weight, and therefore BMI, reduction, and reducing blood glucose levels, as well as HbA1c levels through educational approach and lifestyle changes. The results of those studies point to multicomponent interventions as more effective than single-component interventions.

**Summary:**

An effective workplace intervention aimed to reduce the risk of type 2 diabetes among employees is a multicomponent program consisting of elements such as educational activities, interventions targeting dietary changes and increased physical activity.

**Supplementary Information:**

The online version contains supplementary material available at 10.1007/s11892-023-01521-3.

## Introduction

The International Diabetes Federation estimated in its 2021 report that 537 million adults aged 20–79 were diagnosed with diabetes worldwide, and forecasted the increase to 643 million in 2030, and to 743 million in 2045. The most common type of diabetes is type 2 diabetes, which accounts for more than 90% of all cases of the disease worldwide. In type 2 diabetes, the increase in blood glucose initially results from insulin-resistance, or the inability of the body cells to respond fully to insulin. This type of impairment results in an increase of insulin production, and over time, due to the inability of pancreatic beta cells to keep up with the demand, insulin production becomes insufficient [1].

Appropriate lifestyle modification has been proven effective in preventing or delaying the onset of type 2 diabetes. The main preventive goals should include achieving and maintaining a appropriate body weight, engaging in physical activity for at least 30 min most days of the week, avoiding intake of sugar and saturated fat, and quitting smoking [2].

One of the most well-known programs aimed at preventing type 2 diabetes is the US-led National Diabetes Prevention Program (DPP). This program was initially a multicenter clinical trial. The researched intervention included lifestyle changes in the form of calorie intake reduction and increased physical activity to at least 150 min per week. The results of this study showed that the structured lifestyle change program contributed to weight loss of participants between 5 and 7% of body weight and reduced the risk of developing type 2 diabetes in high-risk adults by 58% [3•]. Currently, the DPP program indicates a framework for diabetes prevention activities, bringing together both public and private sectors. The program partners include federal agencies, state and local health departments, national and community organizations, employers, public and private insurers, health care professionals, academic community education programs and companies that focus on health [4]. An increasing number of employers and insurers in the USA are offering lifestyle change programs for the prevention of type 2 diabetes as health-insuranc-covered benefit [5].

The purpose of this study is to identify and evaluate the effectiveness of interventions aimed at preventing type 2 diabetes that can be implemented in the workplace.

Given the plethora of systematic reviews in the field of diabetes prevention, the authors have decided to conduct an umbrella review for pooled analysis of the available scientific evidence in the field. The methodology utilized in this study allowed for the identification of the highest-level studies in the hierarchy of scientific evidence.

## Material and Method

For this paper, the search of systematic reviews with or without meta-analysis was conducted through Medline (via PubMed), Embase (via OVID) and Cochrane Library databases in accordance to previously prepared strategy (supplementary material). Websites of scientific societies, google scholar, TripDatabase and gray literature were also searched for additional studies or clinical recommendations.

An umbrella review was conducted according to the Cochrane Collaboration guidelines [6]. The search of studies was based on a protocol developed before the work began. It included the criteria for including studies in the review, the search strategy, how studies were selected, and the planned methodology for conducting the analysis and data synthesis. The inclusion criteria for this analysis are cited in the following table (Table 1).

**Table 1:** Systematic review inclusion criteria

Population	General population including both healthy people and diabetes risk-groups (including people with obesity)
Intervention	All interventions in the workplace aimed at diabetes prevention, such as education or life-style changes
Comparator	Not limited
Outcomes	Blood glucose level, glycated hemoglobin level, body weight, BMI and other parameters directly or indirectly indicating risks of diabetes
Research type	Systematic reviews with or without meta-analysis

At every step of the review process studies were selected by two independent analysts (J.Ś., K.W.). All discrepancies were solved by means of consensus together with third author (T.T.).

Out of 603 initially included abstracts, 24 studies were chosen for full-text assessment. The study inclusion criteria were met in 7 studies.

The authors have also found an umbrella review by Proper from 2019 [7], where workplace health-promoting intervention had been assessed. Studies included in this review were aimed at assessment of intervention effectiveness in overweight/obese population and reduction of body-weight (14 studies), psychologically healthy (6 studies), musculoskeletal prophylactics (6 studies) and interventions aimed at metabolic risk factors prophylactics (5 studies). This umbrella study yielded an additional study for our analysis—a 2017 narrative systematic review by Hafez [8] referring to workplace intervention for type 2 diabetes prevention. The 2019 umbrella review by Proper also analyzed the 2009 Conn meta-analysis [9], however, that study had already been included for final assessment based on the full-text assessment.

**Fig. 1:**
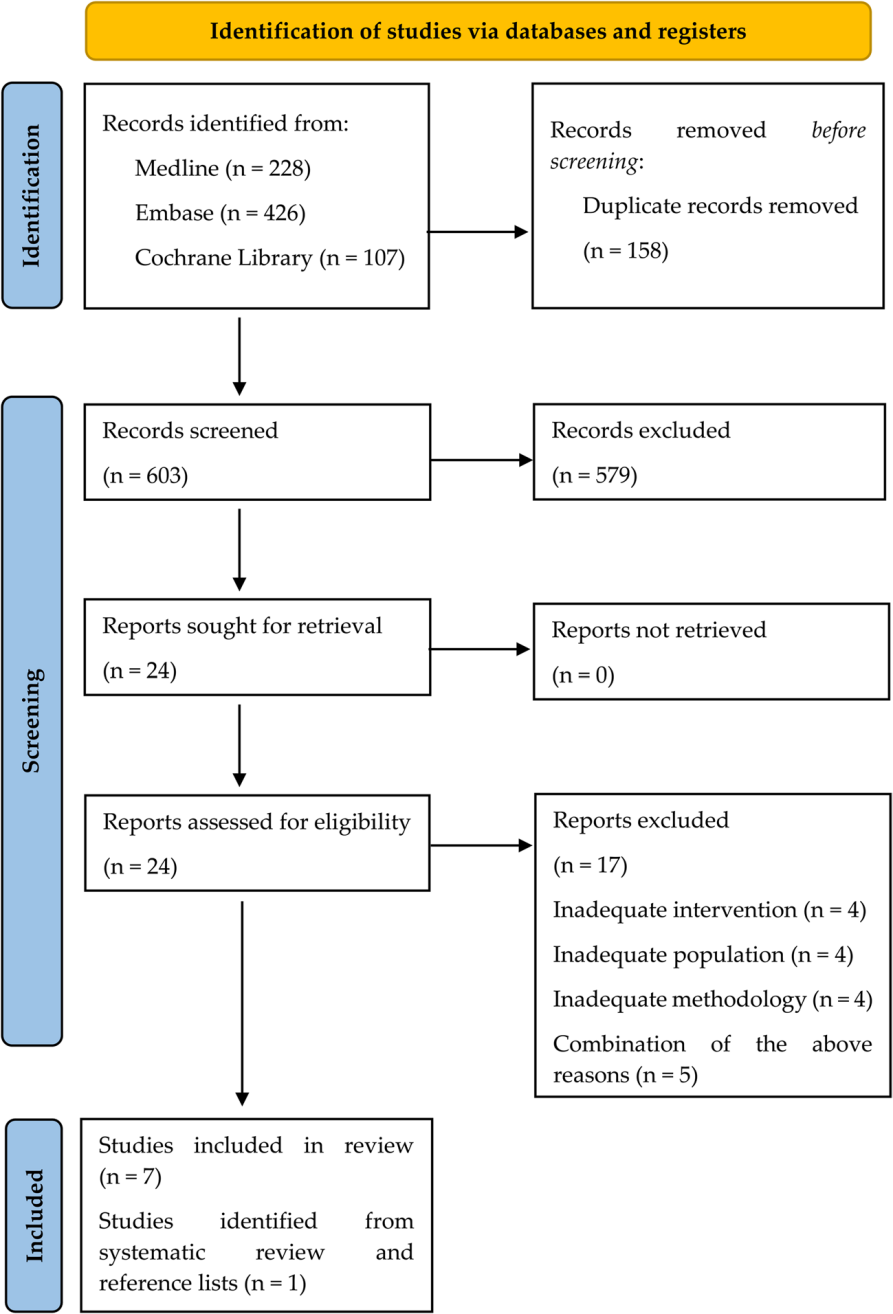
PRISMA flow diagram

The most common exclusion reasons were intervention issues (lack of information about intervention being conducted at a workplace) and methodology (lack of properly described materials, as well as inconsistencies in results description). Figure 1 depicts each step of the selection process. Lists of included and excluded studies are available in the supplementary material.

The quality of the included in the analysis studies was assessed with AMSTAR2 tool [10]. This tool allows for indicating studies of the highest quality; however, it is not including or excluding studies as it is merely a guideline for measuring potential value of results. The highest grade can be awarded to a study in which every questions of the tool yielded positive answers (partial positive is also permissible). A single “no” answer in the critical domain yields study value as “low.” Two or more “no” answers lower the score to “critically low.” The scoring process had been independently performer by two authors (K.W. and J.Ś.). All discrepancies were solved by means of consensus together with third author (T.T.).

## Results

The inclusion criteria for umbrella review encompassing intervention analysis aimed at diabetes prevention at the workplace were met by the following studies (*n* = 7; Fitzpatrick-Lewis 2022, Peñalvo 2021, Inolopú 2019, Brown 2018, Shrestha 2018, Hafez 2017, Conn 2009).

None of the included studies scored high at AMSTAR-2 scale. Most included studies scored a critically low (*n* = 4). Due to the nature of the 2019 Proper study (an umbrella review), the study has been scored according to AMSTAR-2. Detailed analysis results regarding the quality assessment and risk of error analysis have been included in the supplementary material.

### Programs Based on Multicomponent Interventions

According to the results of the 2022 Fitzpatrick-Lewis meta-analysis 2022 conducting in a workplace a structured programs based on US DPP with at least 3 components (health education, diet changes, and increase in physical activity) statistically significantly increases probability body-weight reduction by ≥ 5% (RR = 3.85 [95% CI: (1.58; 9.38)]) and by ≥ 7% (RR = 9.36 [95% CI: (2.31; 37.97)]) in comparison to baseline body-weight among participating employees. Moreover, implementing a DPP-like program statistically significantly influences the decrease of BMI by 0.86 kg/m^2^ (MD = −0.86 [95% CI: (−1.37; −0.34)]) on average. The 4 RCTs analysis also showed statistically significant increase in physical activity among participants—SMD = 0.38 [95% CI: (0.21; 0.55)] [11•].

In the 2021 Peñalvo meta-analysis an influence of multicomponent wellness programs at workplace on anthropometric measurements and blood glucose levels had been assessed. The authors indicated 9 component groups in those programs: screening (A), individual education (B), group education (C), food environment (D), labeling (E); financial incentives (F), physical Activity (G), self-awareness (H), and others (I). The median time of the intervention was 9 months. According to the meta-analysis results the wellness programs consisting of at least single component from A to I statistically significantly influences lowering of the BMI (kg/m^2^) (ES = −0.22 [95% CI: (−0.28; −0.17)]), body-weight (kg) (ES = −0.92 [95% CI: (−1.11; −0.72)]), and waist girth (cm) (ES = −1.47 [95% CI: (−1.96; −0.98)]) in participating in the program employees. For the blood glucose level (mg/dl) outcome the wellness programs (A-I components) the parameter had been statistically significantly lowered—ES = −1.81 [95% CI: (−3.33; −0.28)]. The remaining results of the utilized intervention for percentage body fat reducing (%) (ES = −0.80 [95% CI: (−1.80; 0.21)]; A, B, C, D, F, G, H, I components), decreasing the waist-hip ratio (ES = 0.00 [95% CI: (−0.01; 0.00)]; A, B, C, D, F, G components), as well as increase in fat-free body mass (kg) (ES = 1.01 [95% CI: (−0.82; 2.83)]; B, C, G, I components) were not statistically significant [12•].

In the 2019 Inolopú systematic review, the inclusion criteria were met by 10 studies analyzing interventions aimed at preventing the risk factors and type 2 diabetes at workplace. The studies included 7 analyzed conventional lifestyle changes, 2 analyzed online lifestyle coaching, and 1 analyzed dietary coaching. In 8 studies the interventions were based on the DPP (Diabetes Prevention Program), FDPS (Finnish Diabetes Prevention Study), LiSM10! (Life Style Modification Program for Physical Activity and Nutrition program), and NICE guidelines (National Institute for Health and Care Excellence), as well as JDS/ADA (Japan Diabetes Society and the American Diabetes Association). For outcomes, reduction in body-weight, calorie intake, and 2-h post-prandial blood glucose were chosen. According to 6 included studies utilizing at workplace structured programs based on DPP, FDPS, LiSM10!, and NICE guidelines influenced body-weight reduction among employees. Moreover, the dietary interventions based on JDS/ADA guidelines influenced calorie intake and lowering of the 2-h postprandial blood glucose among the employees (1 study). Two of the included studies were aimed at management and treatment of the previously diagnosed diabetes had not shown influence on the analyzed outcomes [13•].

Contrary, in the 2018 Brown systematic review analyzing the influence of prophylactics programs at workplace aimed at diabetes prevention 8 studies were based on DPP (lifestyle parts) and 14 included other prophylactics programs aimed at improvement of nutritional habits, increasing physical activity and/or management diabetes/cardiovascular disease risk factors. The analyzed studies had shown influence on body-weight and BMI reduction among employees (15 out of 20 studies). Additionally, in 6 out of 10 included in the review studies the influence on HbA1c lowering had been shown [14].

The narrative 2017 Hafez systematic review assessed the efficacy of conducting of prophylactics programs aimed at preventing diabetes at workplace included 10 DPP-based studies and 3 with lower than DPP intensity level aimed at life-style changes support through educational sessions, individual consultations, and websites. The results showed DPP-based programs to influence the reduction of body-weight among employees during 3–6 months (range from −0.4 to −5.1 kg; 8 studies), and 7–12 months (range from −1.43 to −4.9 kg; 6 studies). Moreover, the DPP-based interventions influenced HbA1c lowering (2 studies) and fasting blood glucose level (1 study). The authors had shown only slight influence of the non-DPP-based programs on the body-weight reduction after 6–12 months, as well as on the HbA1c level (2 studies). Conversely, a study showed the ineffectiveness of non-DPP-based program with an observed increase of blood glucose levels 2.5 years after the intervention [8].

### Programs Based on Single-Component Interventions

In the 2018 Shrestha meta-analysis, the influence of the dietary interventions conducted on employees at the workplace on the levels of HbA1c and fasting blood glucose was assessed. The dietary interventions were conducted in small group sessions (8 studies), individual coaching (9 studies) and through setting short-term and long-term heath goals. The results of the meta-analysis of 10 studies showed statistically significant influence of dietary intervention on lowering of the HbA1c (%)—mean change = −0.18% [95% CI: (−0.29; −0.06)]. Conversely, meta-analysis of 12 studies showed no statistically significant difference in fasting blood glucose levels in employees—mean change = −2.60 mg/dl [95% CI: (−5.27; 0,08)] [15].

Contrarywise, in the 2009 Conn meta-analysis the authors had assessed the efficacy of physical activity-related intervention on diabetes risk, fasting blood glucose levels and anthropometric measurement of the employees. Most of the included studies encompassing physical activity programs focused on the motivational/educational sessions (80% of the studies) and supervised exercises conducted at workplace (27% of the studies). The intervention efficacy was measured by size effect for comparing two groups. According to the meta-analysis results, the intervention had positive influence on reducing the diabetes risks, showing the size effect for two-group comparison ranged from 0.90 to 0.98 (two-group post-test = 0.98 [95% CI: (0.06; 1.90)]; two-group pre-post = 0.90 [95% CI: (0.27; 0.53)]; 6 groups), where the size effect had been influenced by small sample size. The mean size effect for diabetes risk had been −12.6 mg/dl for fasting blood glucose levels. Moreover, the intervention had also positively influenced reduction of anthropometric parameters (BMI, weight, abdominal girth, and body fat %), indicating the size effect for two-group post-test at 0.08 [95% CI: (0.02; 0.15); 44 groups] [9].

The detailed characteristics and results of the studies on the prophylactics programs aimed at preventing type 2 diabetes conducted at the workplace have been shown in Table 2.

**Table 2:** Summary and intervention characteristics of the included studies

Author/Year Funding	*N* studies	Population [*N*] Worksite	Intervention (I)	Comparator (C)	Outcomes	Study findings
Fitzpatrick-Lewis 2022 [11] (MA) *Diabetes Canada*	5 RCT	Adult employees in the T2DM risk group T2DM [*N* = 1494] Private corporations; university; railroad company; government agency	DPP-based program or a program utilizing 3 components (health education, diet changes, and increased physical activity) in a workplace	No intervention or delayed/scaled down intervention	After 4–6 months: • 5% or 7% reduction in body-weight • BMI • Physical activity level	The intervention influence the body-weight reduction by ≥ 5% (RR = 3.85 [95% CI: (1.58; 9.38); 4 RCT; *n*/*N* = 84/473 (I); 15/309 (C)]) and by ≥ 7% (RR = 9.36 [95% CI: (2.31; 37.97); 2 RCT; *n*/*N* = 27/90 (I); 2/62 (C)]) in comparison to baseline participants body-weight. The DPP-based program implementation influences lowering of the participants’ BMI–MD = −0.86 (kg/m2) [95%CI: (−1.37; −0.34); 5 RCT; *N* = 521 (I); 348 (C)]. Increase in physical activity levels had been noted among the program participants—SMD = 0.38 [95% CI: (0.21; 0.55); 4 RCT; *N* = 361 (I); 219 (C)].
Peñalvo 2021 [12] (MA) *No funding*	82 RCT, 39 quasi-experimental	Adult employees in the risk group [median = 413] Factories, offices, hospitals, schools (employees), mixed settings	Multicomponent programs in the workplace comprising of at least of the following: Screening (A); Individual education (B); Group education (C); Food environment (D); Labeling (E); Financial incentives (F); Physical Activity (G); Self-awareness (H); Others (I)	Usual care or lesser intensity intervention (i.e., exclusively education on healthy diet)	Median intervention time: 9 months (4.5–18.0) • Changes in adiposity (body weight, BMI, waist circumference, skinfold, body fat percentage) • Biomarker changes glucose and insulin	The efficacy analysis of the wellness programs comprised of every component (A-I) showed positive influence on lowering BMI, body-weight and waist circumference among the participants (ES): • BMI (kg/m^2^) = −0.22 [95% CI: (−0.28; -0.17); 57 studies/67 groups; *N* = 92,698]; • Body-weight (kg) = −0.92 [95% CI: (−1.11; −0.72); 47 studies/59 groups; *N* = 162,019]; • Waist girth (cm) = −1.47 [95% CI: (−1.96; −0.98) (31 studies/37 groups; *N* = 21,334]. Wellness programs influence lowering of the fasting plasma glucose (mg/dl) (ES) = −1.81 [95% CI: (−3.33; −0.28); 21 studies/26 groups; *N* = 30,293]. Showed no influence on decreasing both the percentage fat content (the amount of fat contained in food products) (analyzed components: A, B, C, D, F, G, H, I), nor waist-hip ratio (analyzed components A, B, C, D, F, G) (ES): • Fat content (%) = −0.80% [95% CI: (−1.80; 0.21); (11 studies/13 groups; *N* = 1318); • Waist-hip ratio = 0.00 [95% CI: (−0.01; 0.00) (6 RCT/8 groups; *N* = 2839). Wellness programs comprising programs B, C, G, I components do not influence lowering the non-fat body-weight (kg) = 1.01 [95% CI: (−0.82; 2.83); 4 RCT; *N* = 437].
Inolopú 2019 [13] (SR) *Instituto de Evaluación de Tecnologías en Salud e Investigación* *Fogarty International Center of the US National Institutes of Health*	6 RCT, 4 quasi-experimental	Adult employees in the T2DM risk group or prediabetic [*N* = 2536] Pharmaceutical company; IT firm, college; municipal offices, airline company; finance firm; nursing technicians; not specified (3 studies)	Interventions aimed at preventing risk factors and T2DM occurrence in the workplace: • Conventional lifestyle changing interventions (7); • Lifestyle changes coaching online (2); • Dietary coaching (1). 9 out of 10 included studies based their programs on: • Diabetes Prevention Program DPP (*n* = 3); • Finnish Diabetes Prevention Study (FDPS) (*n* = 2); • Life Style Modification Program for Physical Activity and Nutrition Program (LiSM10!) (*n* = 1); • Guidelines from The National Institute for Health and Care Excellence – NICE (*n* = 1); • Japanese Diabetes Society and the American Diabetes Association (JDS/ADA) (*n* = 1); • Diabetes management: healthy living with diabetes program (*n* = 1); not specified (*n* = 1)	Not specified	4 to 36 months (mean 14,4 months) • Body-weight reduction • Calorie intake • Blood glucose levels 2h postprandial	Structured programs based on DPP, FDPS, LiSM10! and NICE recommendations influence the body-weight reduction (6 studies). In DPP-based 2 studies noted higher percentage in body-weight reduction in the intervention group had been noted: • Among pharmaceutical company employees 5% body-weight reduction in comparison to the pre-intervention baseline had been noted (45% (I) vs. 7% (C); *N* = 89; observation period = 12 months); • Among college employees 7% body-weight reduction in comparison to pre-intervention baseline had been noted (32.4% (I) vs 2.9% (C), *p* < 0.01; *N* = 69; observation period = 7 months). The economic incentives exchanged for body-weight reduction in the DPP-based program influenced body-eight and BMI reduction among nursing technicians (*N* = 99). Nutritional coaching based on JDS/ADA guidelines influences calorie intake reduction and lowering blood glucose level 2-h postprandial (1 study). No influence of the programs focused on management and treatment of diabetes (2 studies).
Brown 2018 [14] (SR) *No information*	6 RCT, 5 quasi-experimental, 11 one-group pre-post study	Adult employees in the T2DM risk group/prediabetic or T2DM diagnosed [*N* = 30,974] Corporate companies; healthcare providers (hospitals, clinics, health departments); insurance companies; manufacturers; universities	Conducting prophylactics programs aimed at worksite diabetes occurrence prevention (the interventions utilized in the programs lasted on average 12–24 weeks mostly conducted as 1 h weekly session with control consultation once or twice a month): • DPP-based programs (lifestyle part): group sessions in groups under 20 employees, most commonly at the cafeteria during lunchtime and during working hours (8 studies); • Remaining programs were aimed at improving nutritional habits, increasing physical activity and/or diabetes management/ cardiovascular risk factors (stress reduction, advice adherence, maintaining body-mass, restricting or quitting stimulants) (14 studies). Medical personnel responsible for conducting the interventions: dietitians, MDs, nurses, as well as psychologists, physiotherapists, and pharmacists (18 studies). Intervention form: online, phone calls, DVD, e-mails, websites, interactive notice boards, mobile apps; pedometers and computer software for physical exercise.	No intervention or delayed/reduced intervention range	After 6–12 months: • Body-mass reduction • BMI • HbA1c levels • Blood pressure	Interventions aimed at diabetes prophylactics influenced reduction in BMI/body-mass (15 out of 20 studies). Prophylactics programs aimed at lowering HbA1c levels (6 out of 10 studies) as well as blood pressure improvement (6 out of 14 studies).
Shrestha 2018 [15] (MA) *NIH Director’s Pioneer Award*	10 RCT, 7 pre-post design studies	Adult employees in CDV risk group (10 studies); diabetic employees (4 studies); employees without confirmed diagnosis or risk factors (3 studies) [*N* = 14,272]	Dietary interventions conducted as part of: group sessions (health educators) (8 studies); individual coaching (face-to-face o rover a phone – 9 studies); setting short-term and long-term health goals. Interventions were conducted from 3 to 36 months (median 12 months) and median of numbers of dietary interventions was 7.	Not specified	• HbA1c levels • Fasting blood glucose levels	Dietary interventions conducted in a workplace influenced HbA1c level by (mean change): −0.18% [95% CI: (−0.29; −0.06) *p* < 0.001; 10 studies). No influence of the dietary intervention on fasting blood glucose levels (mean change): −2.60 mg/dl [95% CI: (−5.27; 0.08) *p* = 0.06; 12 studies).
Hafez 2017 [8] (narrative SR) *No information*	2 RCT, 1 non- RCT, 1 cluster RCT, 3 single group time series, 2 single group pre-post, 1 multi-group pre-post, 3 cohort studies	Adult employees from T2DM risk group [*N* enrolled = 3746; *N* analyzed = 1317] Departments of public hospital, newspaper publisher and city/county health department/county police; manufacturer of medical technologies; county employees; maintenance facility; nursing home facility employees; manufacturing plant; pharmaceutical company; organizations; university; 5 companies: health insurance, wharf, camper, food industry, medical equipment supplier	Conducting prophylactics programs in workplace aimed at diabetes prevention: DPP-based programs (10 studies): • In 6 out of 10 at least 16 basic DPP sessions had been conducted and retaining phase had been utilized; • In 2 studies the time-frame for the sessions had been shortened from 16 to 12 or 4 weeks; • The sessions were most commonly held during lunch or after working hours (4 studies); • in 2 studies the participants were allowed to choose suitable form and time for their interventions; • in 6 studies financial incentives had been offered for registering and participating in the program. Non-DPP-based programs: lower intensity than in DPP-based programs aimed at support in lifestyle changes through educational sessions, websites and individual consultations (3 studies).	No intervention or delayed/reduced intervention range	After 3–12 months: • Body-mass reduction • HbA1c levels • Fasting blood glucose levels	DPP-based programs influenced: • Body-weight reduction by −0.4 to −5.1 kg in 3–6 months (8 studies) and by −1.43 to −4.9 kg in 7–12 months (6 studies); • Percentage body-weight reduction by −0.5 to −5.5% in 12 weeks to 12 months (7 studies); • Body-weight reduction by at least 5% of the baseline body-weight after 16 weeks since the intervention (14–56% of participants; 4 studies); • Decreased HbA1c levels (2 studies) and fasting blood glucose levels (1 study). Non DPP-based programs slightly influenced body-weight reduction after 6–12 months and HbA1c value decrease (2 studies). In 1 study the increase of blood glucose level had been noted after 2.5 years after the intervention (0.27 mmol/l among men and 0.35 mmol/l among women).
Conn 2009 [9] (MA) *No funding*	206 comparisons/ 138 varied design studies (pre, post, pre-post studies)	Adult employees from revenue-oriented companies (55 studies) or non-profit organizations (50 studies)—mainly educational institutions, healthcare providers, governmental offices, and manufacturers [*N* = 38,231]	Physical activity programs in a workplace in a form of supervised exercises (27% of studies) and/or motivational/educational sessions (80% of studies)	Two-groups pre-post intervention	• Diabetes risk • Fasting blood glucose • Anthropometric measures (encoded as BMI, weight, abdominal girth, body fat)	The intervention influenced positively decreased risk of diabetes, with a size effect between groups between 0.90 and 0.98—two-group post-test = 0.98 [95% CI: (0.06; 1.90); two-group pre-post = 0.90 [95% CI: (0.27; 0.53); 6 groups. The size effect was influenced by small number of studies/sample. Mean size effect for diabetes risk had been −12.6 mg/dl for fasting blood glucose—treatment group/control subjects (81.0 (I) vs 93.6 (C)). The intervention has influenced positively lowering of the anthropometric parameters with a size effect for two-group post-test = 0.08 [95% CI: (0.02; 0.15)]; 44 groups.

*BMI*, body mass index; *CI*, confidence interval; *C*, comparator; *CVD*, cardiovascular disease; *I*, intervention; *DPP*, Diabetes Prevention Program; *ES*, effect size (inverse-variance random-effects meta-analysis to estimate an overall summary effect size [95%CI]); *FDPS*, Finnish Diabetes Prevention Study; *HbA1c*, glycated hemoglobin; *JDS/ADA*, Japan Diabetes Society and the American Diabetes Association; *LiSM10!*, Life Style Modification Program for Physical Activity and Nutrition program; *MA*, meta-analysis; *MD*, mean difference; *N*, number; *NICE*, National Institute for Health and Care Excellence; *RCT*, randomized controlled trial; *RR*, relative risk; *SMD*, standardized mean difference; *SR*, systematic review; *T2DM*, type 2 diabetes mellitus

## Discussion

In the study, the analysis of the conducted at workplace interventions aimed at diabetes prevention was made. Most (4 out of 7) included in the review studies were based on the US Diabetes Prevention Program (DPP) or other, similar programs [8, 11•, 13•, 14]. A single study assessed conducting multicomponent wellness programs at workplace and its influence on the anthropometric parameters, as well as the blood glucose levels [12•]. The use of single-component interventions (i.e., dietary interventions [15], physical activity-related [9] had also been noted.

It should be noted that the type 2 diabetes risk is related to another significant health issue—the obesity [16–19]. Therefore the actions aimed at reducing body-weight may also influence diabetes risk reduction. To that effect the analysis presented in this paper had been not only of the parameters directly related to diabetes (i.e., fasting blood glucose levels or glycated hemoglobin), but of those indirectly related also (i.e., body-weight and BMI changes).

According to results of the found studies the interventions conducted at workplace can be an efficient diabetes risk reducing method. The best results were noted for interventions comprising several components, i.e., health education, diet changes or increase in physical activity [8, 11•, 12•, 13•]. The key parameters of the physical activity seem to be the intensity and longevity of the intervention (the longer and the more intensive, the better results of the analyzed parameters).

Among different types of programs, the most common are those based on DPP. Every study analyzing DPP-based programs achieved statistically significant results representing improvement of parameters such as physical activity levels, blood pressure, body-weight, BMI, HbA1c levels, and fasting blood glucose levels [8, 11•, 13•, 14]. In the 2017 Hafez study the authors had analyzed and compared non-DPP-based programs (encompassing lower than DPP intensity interventions aimed at supporting life-style changes through educational sessions, websites, and individual consultations) described in 3 studies [8]. In 2 out of 3 studies those interventions only slightly influenced body-mass reduction after 6–12 months and decrease in HbA1c levels [20, 21], while the remaining study showed an increase in blood glucose levels 2.5 years after the intervention [22].

When single component interventions were utilized separately, the effects were much smaller or inconclusive (i.e., some parameters showed improvement, while others did not change). One such example was a 2018 Shrestha study, where utilized dietary interventions in form of group or individual sessions (mean time of intervention—12 months) noted slight decrease in HbA1c levels, yet no statistical significant results were obtained for fasting blood glucose level [15].

In most of the studies found, the observation period was about 12 months. Such time is not long enough to make a clear statement about the effectiveness of the measures. It seems necessary to carry out further analyses showing the size effect after several years after the implementation of the interventions. Longevity is one of the key elements for assessing the effectiveness of interventions.

For the purpose of the discussion, current recommendations on diabetes prevention were reviewed. They referred to the types and ways of implementing interventions in people in diabetes risk groups, including overweight and obesity, low levels of physical activity, cases of diabetes in the family and the pre-diabetic state. According to recommendations, the aforementioned factors can occur both separately and simultaneously [23–29].

As part of preventive measures aimed at type 2 diabetes, it is recommended to implement broad educational activities focused on making the patient aware of the health risks associated with type 2 diabetes [23, 27, 29, 30]. It is also recommended to implement interventions aimed at lifestyle modification, including limiting the intake of products increasing the risk of type 2 diabetes (including fats, simple sugars, and sweetened beverages), while increasing products showing a preventive effect on the disease. Moreover, if it is deemed necessary, specific dietary patterns such as the DASH diet or the Mediterranean diet should be recommended [25, 27, 29, 31–33]. Increasing the level of physical activity among people at risk for type 2 diabetes should also be an important part of an intervention aimed at lifestyle modification. The main goal of encouraging the introduction of physical activity should be to reduce body weight and increase energy expenditure, especially in overweight or obese individuals [23, 25, 27–29, 32, 33]. Ultimately, it is recommended for people at risk for type 2 diabetes to engage in moderate-intensity physical activity tasks (e.g., volleyball, tennis, intermediate or long-distance running) at a minimum of 150 min per week [23, 25, 28, 32].

The recommendations also emphasize the need for implementation of screening aimed at type 2 diabetes. The included recommendations currently recommend the use of fasting blood glucose measurement, oral glucose tolerance test and blood glucose measurement as target screening tools. These technologies, according to the recommendations, can be used interchangeably, and the age for starting regular screening in the most recent documents is set at 35 years [23–25].

Some guidelines also emphasize the importance of conducting activities in the workplace [27, 29]. The National Institute for Health and Clinical Excellence recommendations emphasize that awareness-raising activities should be carried out in multiple locations. In addition to the workplace, these could include primary care facilities, pharmacies, dental offices, offices, stores, libraries, nursing homes, assisted living centers, or churches. The document also stresses that comprehensive programs should be conducted in the easily accessed workplace (at various times of the day) [27]). Conversely, another European recommendation indicates that behavioral interventions aimed at diabetes prevention may be conducted in health care facilities, the workplace, and the participant's home [29].

## Review Limitations

The review included exclusively publications in English. The studies included in the systematic reviews found analysed a diverse population in terms of health status. Some studies analyzed pre-diabetic or diagnosed diabetic population, while others looked at the impact of the intervention on healthy adults. The studies also included different endpoints, intervention time and follow-up period. They used varied methods of data presentation. The aforementioned elements created a considerable heterogeneity among the studies, which in some cases made a meta-analysis impossible. As a result, it is also not possible to make simple comparisons of results between publications.

The review focused only on parameters that are directly or indirectly related to the incidence of diabetes. Sometimes the included publications analyzed a broader spectrum of measures and endpoints (e.g., relating to cholesterol levels). However, for the sake of data transparency and clarity, the authors deemed it impossible to describe them all in detail.

## Conclusions

Based on available studies, it is possible to conclude that multicomponent programs that include elements such as educational activities, interventions directed at changing diet and increasing physical activity are effective in reducing the risk of type 2 diabetes. Multicomponent interventions are more effective than those undertaken separately. Therefore, multicomponent interventions based on best practices and global recommendations should be implemented in the workplace first and foremost, with appropriate intensity and duration (a minimum of several months). An example would be programs based on the National Diabetes Prevention Program implemented in the USA.

Activities undertaken as part of programs aimed at diabetes prevention simultaneously have a positive impact on a number of other diseases by, for example, reducing body weight or lowering the risk of cardiovascular disease. The benefits of introducing health-promoting measures are therefore undeniable, and should be considered both for people at risk for diabetes (to reduce their risk levels), as well as for healthy people, who will be able to improve their knowledge of diabetes and make health-promoting changes in their daily lives.

Due to the relatively short follow-up period of the studies found, further analyses and high-quality RCTs would need to be conducted to assess the durability of the effect over time, several years after its application.

### Supplementary Information

ESM 1 (DOCX 35 kb)

## Data Availability

Not applicable.
