# Supplementary material for: Workplace Interventions for Type 2 Diabetes Mellitus Prevention—an Umbrella Review
Source: Curr Diab Rep. 2023 Sep 20;23(10):293–304. doi: 10.1007/s11892-023-01521-3 (PMC10520112; doi:10.1007/s11892-023-01521-3)
Supplement: Supplementary file 1 — (DOCX 35 kb) [file 11892_2023_1521_MOESM1_ESM.docx]

**Search strategy Medline (via PubMed)**

| **ID** | **Keyword** | **Result** |
| --- | --- | --- |
| #1 | Search: diabetes mellitus[MeSH Terms] | 490468 |
| #2 | Search: diabetes[Title/Abstract] | 630167 |
| #3 | Search: diabetes type 2[Title/Abstract] | 1713 |
| #4 | Search: DMT2[Title/Abstract] | 146 |
| #5 | Search: Noninsulin Dependent Diabetes Mellitus[Title/Abstract] | 886 |
| #6 | Search: T2D[Title/Abstract] | 14971 |
| #7 | Search: ((((("Diabetes Mellitus"[Mesh]) OR (diabetes[Title/Abstract])) OR (diabetes type 2[Title/Abstract])) OR (DMT2[Title/Abstract])) OR ("Noninsulin Dependent Diabetes Mellitus"[Title/Abstract])) OR (T2D[Title/Abstract]) | 758927 |
| #8 | Search: workplace[Mesh] | 28440 |
| #9 | Search: workplace[Title/Abstract] | 47097 |
| #10 | Search: work-based[Title/Abstract] | 2177 |
| #11 | Search: work[Title/Abstract] | 1233445 |
| #12 | Search: office[Title/Abstract] | 71154 |
| #13 | Search: occupational[Title/Abstract] | 147344 |
| #14 | Search: (((((workplace[Mesh]) OR (workplace[Title/Abstract])) OR (work-based[Title/Abstract])) OR (work[Title/Abstract])) OR (office[Title/Abstract])) OR (occupational[Title/Abstract]) | 1434181 |
| #15 | Search: health promotion[Mesh] | 84032 |
| #16 | Search: health promotion[Title/Abstract] | 40126 |
| #17 | Search: program*[Title/Abstract] | 1045192 |
| #18 | Search: intervention*[Title/Abstract] | 1253054 |
| #19 | Search: policy[Title/Abstract] | 249579 |
| #20 | Search: policie*[Title/Abstract] | 119963 |
| #21 | Search: prevention[Title/Abstract] | 674179 |
| #22 | Search: ((((((health promotion[Mesh]) OR (health promotion[Title/Abstract])) OR (program*[Title/Abstract])) OR (intervention*[Title/Abstract])) OR (policy[Title/Abstract])) OR (policie*[Title/Abstract])) OR (prevention[Title/Abstract]) | 2895 391 |
| #23 | Search: ((((((workplace[Mesh]) OR (workplace[Title/Abstract])) OR (work-based[Title/Abstract])) OR (work[Title/Abstract])) OR (office[Title/Abstract])) OR (occupational[Title/Abstract])) AND (((((((health promotion[Mesh]) OR (health Promotion[Title/Abstract])) OR (program*[Title/Abstract])) OR (intervention*[Title/Abstract])) OR (policy[Title/Abstract])) OR (policie*[Title/Abstract])) OR (prevention[Title/Abstract])) | 229062 |
| #24 | Search: ((((((diabetes mellitus[Mesh]) OR (diabetes[Title/Abstract])) OR (diabetes type 2[Title/Abstract])) OR (DMT2[Title/Abstract])) OR (Noninsulin Dependent Diabetes Mellitus[Title/Abstract])) OR (T2D[Title/Abstract])) AND (((((((workplace[Mesh]) OR (workplace[Title/Abstract])) OR (work-based[Title/Abstract])) OR (work[Title/Abstract])) OR (office[Title/Abstract])) OR (occupational[Title/Abstract])) AND (((((((health promotion[Mesh]) OR (Health Promotion[Title/Abstract])) OR (program*[Title/Abstract])) OR (intervention*[Title/Abstract])) OR (policy[Title/Abstract])) OR (policie*[Title/Abstract])) OR (prevention[Title/Abstract]))) | 5471 |
| #25 | Search: ((((((diabetes mellitus[Mesh]) OR (diabetes[Title/Abstract])) OR (diabetes type 2[Title/Abstract])) OR (DMT2[Title/Abstract])) OR (Noninsulin Dependent Diabetes Mellitus[Title/Abstract])) OR (T2D[Title/Abstract])) AND (((((((workplace[Mesh]) OR (workplace[Title/Abstract])) OR (work-based[Title/Abstract])) OR (work[Title/Abstract])) OR (office[Title/Abstract])) OR (occupational[Title/Abstract])) AND (((((((health promotion[Mesh]) OR (Health Promotion[Title/Abstract])) OR (program*[Title/Abstract])) OR (intervention*[Title/Abstract])) OR (policy[Title/Abstract])) OR (policie*[Title/Abstract])) OR (prevention[Title/Abstract]))) Filters: Meta-Analysis, Systematic Review | 228 |

**Search strategy Cochrane**

| **ID** | **Keyword** | **Result** |
| --- | --- | --- |
| #1 | MeSH descriptor: [Diabetes Mellitus] explode all trees | 35898 |
| #2 | (diabetes):ti,ab,kw | 108860 |
| #3 | (diabetes type 2):ti,ab,kw | 55630 |
| #4 | (DMT2):ti,ab,kw | 63 |
| #5 | (Noninsulin Dependent Diabetes Mellitus):ti,ab,kw | 634 |
| #6 | (T2D):ti,ab,kw | 4103 |
| #7 | #1 OR #2 OR #3 OR #4 OR #5 OR #6 | 109327 |
| #8 | MeSH descriptor: [Workplace] explode all trees | 988 |
| #9 | (workplace):ti,ab,kw | 3538 |
| #10 | (work-based):ti,ab,kw | 106 |
| #11 | (work):ti,ab,kw | 74096 |
| #12 | (office):ti,ab,kw | 12040 |
| #13 | (occupational):ti,ab,kw | 14664 |
| #14 | #8 OR #9 OR #10 OR #11 OR #12 OR #13 | 95640 |
| #15 | MeSH descriptor: [Health Promotion] explode all trees | 7187 |
| #16 | (health promotion):ti,ab,kw | 28121 |
| #17 | (program*):ti,ab,kw | 147344 |
| #18 | (intervention*):ti,ab,kw | 512669 |
| #19 | (policy):ti,ab,kw | 11721 |
| #20 | (policie*):ti,ab,kw | 2880 |
| #21 | (prevention):ti,ab,kw | 257882 |
| #22 | #15 OR #16 OR #17 OR #18 OR #19 OR #20 OR #21 | 743754 |
| #23 | #14 AND #22 | 55208 |
| #24 | #7 AND #23 | 3163 |
| #25 | #7 AND #23 in Cochrane Reviews | 107 |

**Search strategy Embase (via Ovid)**

| **ID** | **Keyword** | **Result** |
| --- | --- | --- |
| 1 | exp diabetes mellitus/ | 1084259 |
| 2 | diabetes.ab,kw,ti. | 908106 |
| 3 | "diabetes type 2".ab,kw,ti. | 3174 |
| 4 | DMT2.ab,kw,ti. | 366 |
| 5 | "Noninsulin Dependent Diabetes Mellitus".ab,kw,ti. | 973 |
| 6 | T2D.ab,kw,ti. | 27010 |
| 7 | 1 or 2 or 3 or 4 or 5 or 6 | 1239831 |
| 8 | exp workplace/ | 51252 |
| 9 | workplace.ab,kw,ti. | 54705 |
| 10 | work-based.ab,kw,ti. | 2490 |
| 11 | work.ab,kw,ti. | 1460718 |
| 12 | office.ab,kw,ti. | 93304 |
| 13 | occupational.ab,kw,ti. | 155530 |
| 14 | 8 or 9 or 10 or 11 or 12 or 13 | 1692317 |
| 15 | exp health promotion/ | 108887 |
| 16 | "health promotion".ab,kw,ti. | 45692 |
| 17 | "program* ".ab,kw,ti. | 1273016 |
| 18 | "intervention* ".ab,kw,ti. | 1694737 |
| 19 | policy.ab,kw,ti. | 268631 |
| 20 | "policie* ".ab,kw,ti. | 131843 |
| 21 | prevention.ab,kw,ti. | 795943 |
| 22 | 15 or 16 or 17 or 18 or 19 or 20 or 21 | 3620930 |
| 23 | 14 and 22 | 303343 |
| 24 | 7 and 23 | 10831 |
| 25 | limit 24 to (meta analysis or "systematic review") | 426 |

**Table 1. List of studies included and excluded after full-text analysis**

| **Lp.** | **Authors, Title, Journal** | **Full text status** |
| --- | --- | --- |
| **1.** | **Proper KI, van Oostrom SH. The effectiveness of workplace health promotion interventions on physical and mental health outcomes - a systematic review of reviews. Scand J Work Environ Health. 2019 Nov 1;45(6):546-559. doi: 10.5271/sjweh.3833. Epub 2019 May 28. PMID: 31134284.** | **Included** |
| **2.** | **Brown SA, García AA, Zuñiga JA, Lewis KA. Effectiveness of workplace diabetes prevention programs: A systematic review of the evidence. Patient Educ Couns. 2018 Jun;101(6):1036-1050. doi: 10.1016/j.pec.2018.01.001. Epub 2018 Jan 5. PMID: 29339040.** | **Included** |
| **3.** | **Shrestha A, Karmacharya BM, Khudyakov P, Weber MB, Spiegelman D. Dietary interventions to prevent and manage diabetes in worksite settings: a meta-analysis. J Occup Health. 2018 Jan 25;60(1):31-45. doi: 10.1539/joh.17-0121-RA. Epub 2017 Nov 29. PMID: 29187673; PMCID: PMC5799099.** | **Included** |
| **4.** | **Conn VS, Hafdahl AR, Cooper PS, Brown LM, Lusk SL. Meta-analysis of workplace physical activity interventions. Am J Prev Med. 2009 Oct;37(4):330-9. doi: 10.1016/j.amepre.2009.06.008. PMID: 19765506; PMCID: PMC2758638.** | **Included** |
| **5.** | **Fitzpatrick-Lewis D, Ali MU, Horvath S, Nagpal S, Ghanem S, Sherifali D. Effectiveness of Workplace Interventions to Reduce the Risk for Type 2 Diabetes: A Systematic Review and Meta-Analysis. Can J Diabetes. 2022 Feb;46(1):84-98. doi: 10.1016/j.jcjd.2021.04.003. Epub 2021 Apr 20. PMID: 34053879.** | **Included** |
| **6.** | **Inolopú J, Hilario-Huapaya N, Tantaleán-Del-Águila MA, Hurtado-Roca Y, Ugarte-Gil C. Interventions for the prevention of risk factors and incidence of type 2 diabetes in the work environment: a systematic review. Rev Saude Publica. 2019 Dec 2;53:101. doi: 10.11606/s1518-8787.2019053001084. PMID: 31800912; PMCID: PMC6863112.** | **Included** |
| **7.** | **Peñalvo JL, Sagastume D, Mertens E, Uzhova I, Smith J, Wu JHY, Bishop E, Onopa J, Shi P, Micha R, Mozaffarian D. Effectiveness of workplace wellness programmes for dietary habits, overweight, and cardiometabolic health: a systematic review and meta-analysis. Lancet Public Health. 2021 Sep;6(9):e648-e660. doi: 10.1016/S2468-2667(21)00140-7. PMID: 34454642; PMCID: PMC8627548.** | **Included** |
| 8. | Melián-Fleitas L, Franco-Pérez Á, Caballero P, Sanz-Lorente M, Wanden-Berghe C, Sanz-Valero J. Influence of Nutrition, Food and Diet-Related Interventions in the Workplace: A Meta-Analysis with Meta-Regression. Nutrients. 2021 Nov 4;13(11):3945. doi: 10.3390/nu13113945. PMID: 34836200; PMCID: PMC8622081. | Ecluded (M) |
| 9. | Rachmah Q, Martiana T, Mulyono M, Paskarini I, Dwiyanti E, Widajati N, Ernawati M, Ardyanto YD, Tualeka AR, Haqi DN, Arini SY, Alayyannur PA. The effectiveness of nutrition and health intervention in workplace setting: a systematic review. J Public Health Res. 2021 Nov 15;11(1):2312. doi: 10.4081/jphr.2021.2312. PMID: 34781627; PMCID: PMC8859724. | Excluded (P) |
| 10. | Prince SA, Rasmussen CL, Biswas A, Holtermann A, Aulakh T, Merucci K, Coenen P. The effect of leisure time physical activity and sedentary behaviour on the health of workers with different occupational physical activity demands: a systematic review. Int J Behav Nutr Phys Act. 2021 Jul 20;18(1):100. doi: 10.1186/s12966-021-01166-z. PMID: 34284795; PMCID: PMC8290554. | Excluded (P) |
| 11. | Tong B, Kapanen AI, Yuen J. Third-party Reimbursement of Pharmacist-Led Cardiovascular and Diabetes Preventive Health Services for Workplace Health Initiatives: A Narrative Systematic Review. Innov Pharm. 2021;12(1):10.24926/iip.v12i1.3591. Published 2021 Jan 8. doi:10.24926/iip.v12i1.3591 | Excluded (I) |
| 12. | Shrestha N, Kukkonen-Harjula KT, Verbeek JH, Ijaz S, Hermans V, Pedisic Z. Workplace interventions for reducing sitting at work. Cochrane Database Syst Rev. 2018 Dec 17;12(12):CD010912. doi: 10.1002/14651858.CD010912.pub5. PMID: 30556590; PMCID: PMC6517221. | Excluded (P) |
| 13. | Taylor WC, Suminski RR, Das BM, Paxton RJ, Craig DW. Organizational Culture and Implications for Workplace Interventions to Reduce Sitting Time Among Office-Based Workers: A Systematic Review. Front Public Health. 2018 Sep 24;6:263. doi: 10.3389/fpubh.2018.00263. PMID: 30320051; PMCID: PMC6165892. | Excluded (P, M) |
| 14. | Freak-Poli R, Cumpston M, Albarqouni L, Clemes SA, Peeters A. Workplace pedometer interventions for increasing physical activity. Cochrane Database Syst Rev. 2020 Jul 21;7(7):CD009209. doi: 10.1002/14651858.CD009209.pub3. PMID: 32700325; PMCID: PMC7389933. | Excluded (P) |
| 15. | Nazarov S, Manuwald U, Leonardi M, Silvaggi F, Foucaud J, Lamore K, Guastafierro E, Scaratti C, Lindström J, Rothe U. Chronic Diseases and Employment: Which Interventions Support the Maintenance of Work and Return to Work among Workers with Chronic Illnesses? A Systematic Review. Int J Environ Res Public Health. 2019 May 27;16(10):1864. doi: 10.3390/ijerph16101864. PMID: 31137817; PMCID: PMC6572561. | Excluded (I, M) |
| 16. | Mori K, Mori T, Nagata T, Nagata M, Iwasaki M, Sakai H, Kimura K, Shinzato N. Factors of occurrence and improvement methods of presenteeism attributed to diabetes: A systematic review. J Occup Health. 2019 Jan;61(1):36-53. doi: 10.1002/1348-9585.12034. PMID: 30698334; PMCID: PMC6499359. | Excluded (I) |
| 17. | Mackenzie K, Such E, Norman P, Goyder E. The development, implementation and evaluation of interventions to reduce workplace sitting: a qualitative systematic review and evidence-based operational framework. BMC Public Health. 2018 Jul 4;18(1):833. doi: 10.1186/s12889-018-5768-z. PMID: 29973188; PMCID: PMC6033205. | Excluded (P, M) |
| 18. | Brierley ML, Chater AM, Smith LR, Bailey DP. The Effectiveness of Sedentary Behaviour Reduction Workplace Interventions on Cardiometabolic Risk Markers: A Systematic Review. Sports Med. 2019 Nov;49(11):1739-1767. doi: 10.1007/s40279-019-01168-9. PMID: 31429035. | Excluded (M) |
| 19. | Li W, Yi G, Chen Z, Dai X, Wu J, Peng Y, Ruan W, Lu Z, Wang D. Is job strain associated with a higher risk of type 2 diabetes mellitus? A systematic review and meta-analysis of prospective cohort studies. Scand J Work Environ Health. 2021 May 1;47(4):249-257. doi: 10.5271/sjweh.3938. Epub 2021 Jan 6. PMID: 33404062; PMCID: PMC8091067. | Excluded (M) |
| 20. | Dempsey PC, Biddle SJH, Buman MP, Chastin S, Ekelund U, Friedenreich CM, Katzmarzyk PT, Leitzmann MF, Stamatakis E, van der Ploeg HP, Willumsen J, Bull F. New global guidelines on sedentary behaviour and health for adults: broadening the behavioural targets. Int J Behav Nutr Phys Act. 2020 Nov 26;17(1):151. doi: 10.1186/s12966-020-01044-0. PMID: 33239026; PMCID: PMC7691115. | Excluded (M) |
| 21. | Ramsey Buchanan L, Rooks-Peck CR, Finnie RKC, Wethington HR, Jacob V, Fulton JE, Johnson DB, Kahwati LC, Pratt CA, Ramirez G, Mercer SL, Glanz K; Community Preventive Services Task Force. Reducing Recreational Sedentary Screen Time: A Community Guide Systematic Review. Am J Prev Med. 2016 Mar;50(3):402-415. doi: 10.1016/j.amepre.2015.09.030. Erratum in: Am J Prev Med. 2016 Jun;50(6):809. PMID: 26897342. | Excluded (I) |
| 22. | Neuhaus M, Eakin EG, Straker L, Owen N, Dunstan DW, Reid N, Healy GN. Reducing occupational sedentary time: a systematic review and meta-analysis of evidence on activity-permissive workstations. Obes Rev. 2014 Oct;15(10):822-38. doi: 10.1111/obr.12201. Epub 2014 Jul 11. PMID: 25040784. | Excluded (P, I) |
| 23. | Yang X, Di W, Zeng Y, Liu D, Han M, Qie R, Huang S, Zhao Y, Feng Y, Hu D, Sun L. Association between shift work and risk of metabolic syndrome: A systematic review and meta-analysis. Nutr Metab Cardiovasc Dis. 2021 Sep 22;31(10):2792-2799. doi: 10.1016/j.numecd.2021.06.007. Epub 2021 Jun 21. PMID: 34332862. | Excluded (I, M) |
| 24. | Breton MC, Guénette L, Amiche MA, Kayibanda JF, Grégoire JP, Moisan J. Burden of diabetes on the ability to work: a systematic review. Diabetes Care. 2013;36(3):740-749. doi:10.2337/dc12-0354 | Excluded (I) |

P – population; I – intervention; M – methodology

**AMSTAR2**

The systematic reviews included in the analysis received the following ratings:

- high – none.
- low – Peñalvo 2021, Inolopú 2019, Shrestha 2018;
- critically low – Fitzpatrick-Lewis 2022, Brown 2018, Hafez 2017, Conn 2009.

**Table 2. AMSTAR2 rating**

| **Publication** | **Item 2** | **Item 4** | **Item 7** | **Item 9** | **Item 11** | **Item 13** | **Item 15** | **Overall rating** |
| --- | --- | --- | --- | --- | --- | --- | --- | --- |
| Fitzpatrick-Lewis 2022 (MA) | Partial  Yes | Partial  Yes | No | Yes | Yes | Yes | No | **Critically Low** |
| Peñalvo 2021 (MA) | Yes | Partial  Yes | No | Partial  Yes | Yes | Yes | Yes | **Low** |
| Inolopú 2019 (SR) | Partial  Yes | Partial  Yes | No | Yes | – | Yes | – | **Low** |
| Proper 2019 (UR) | **Not applicable** | | | | | | | |
| Brown 2018 (SR) | No | Partial  Yes | No | Partial  Yes | – | Yes | – | **Critically Low** |
| Shrestha 2018 (MA) | Partial  Yes | Partial  Yes | No | Partial  Yes | Yes | Yes | Yes | **Low** |
| Hafez 2017 (SR) | No | Partial  Yes | No | No | – | No | – | **Critically Low** |
| Conn 2009 (MA) | Partial  Yes | Partial  Yes | No | No | Yes | No | Yes | **Critically Low** |

MA – meta-analysis; SR – systematic review; UR – umbrella review

*Critical domains: item 2 – protocol registered before commencement of the review; item 4 – adequacy of the literaturę search; item 7 – justyfication for excluding individual studies; item 9 – risk of bias from individual studies being included in the review; item 11 – appropriateness of meta-analytical methods; item 13 – consideration of risk of bias when interpreting the results of the review; item 15 – assessment of presence and likely impact of publication bias.*
